# Supplementary figures and images for: Exploring Novel Genomic Loci and Candidate Genes Associated with Plant Height in Bulgarian Bread Wheat via Multi-Model GWAS
Source: Plants (Basel). 2024 Oct 3;13(19):2775. doi: 10.3390/plants13192775 (PMC11479123; doi:10.3390/plants13192775)

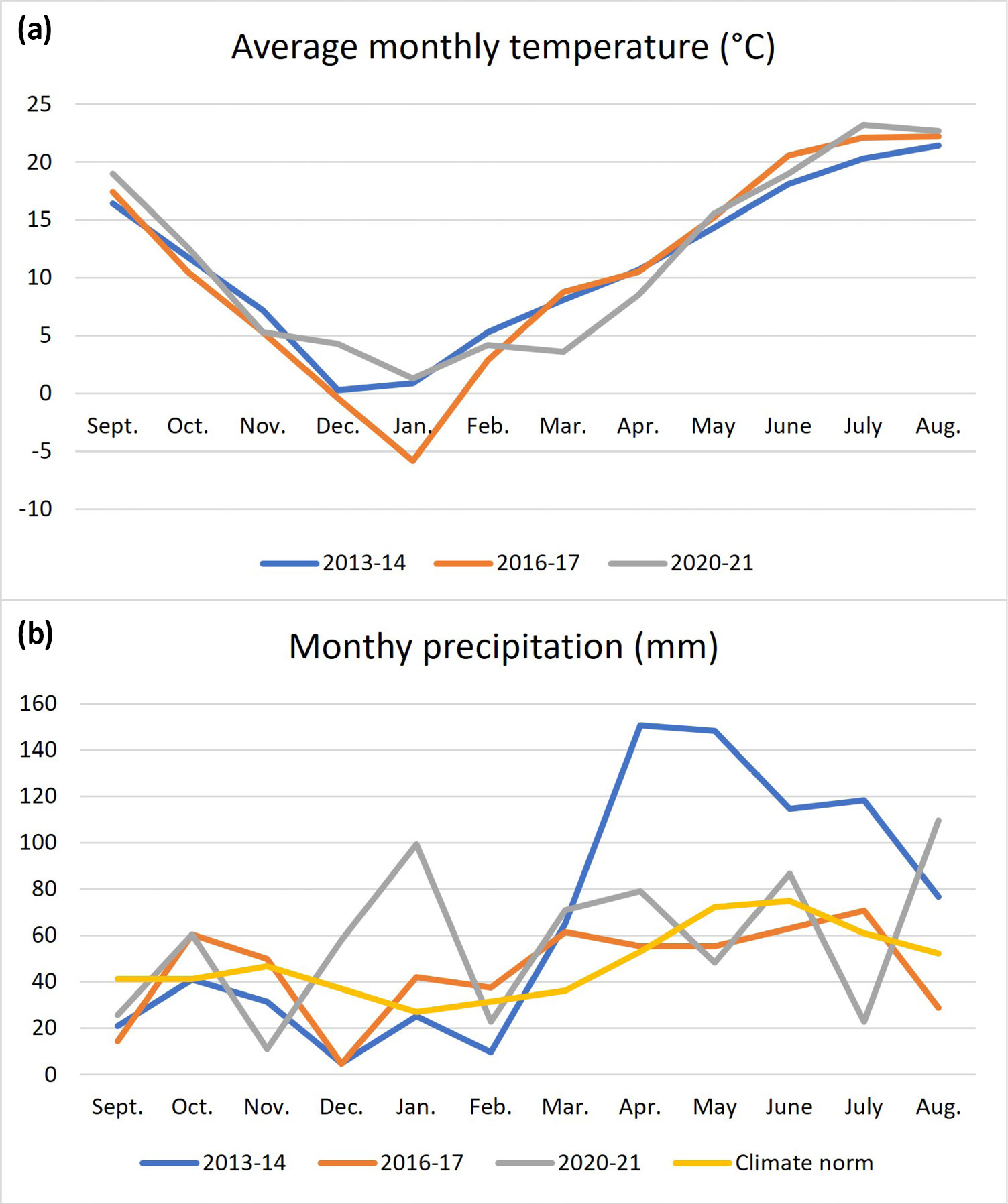

Supplement: Supplementary file 1 [file plants-13-02775-s001.zip › Figure_S1.jpg]

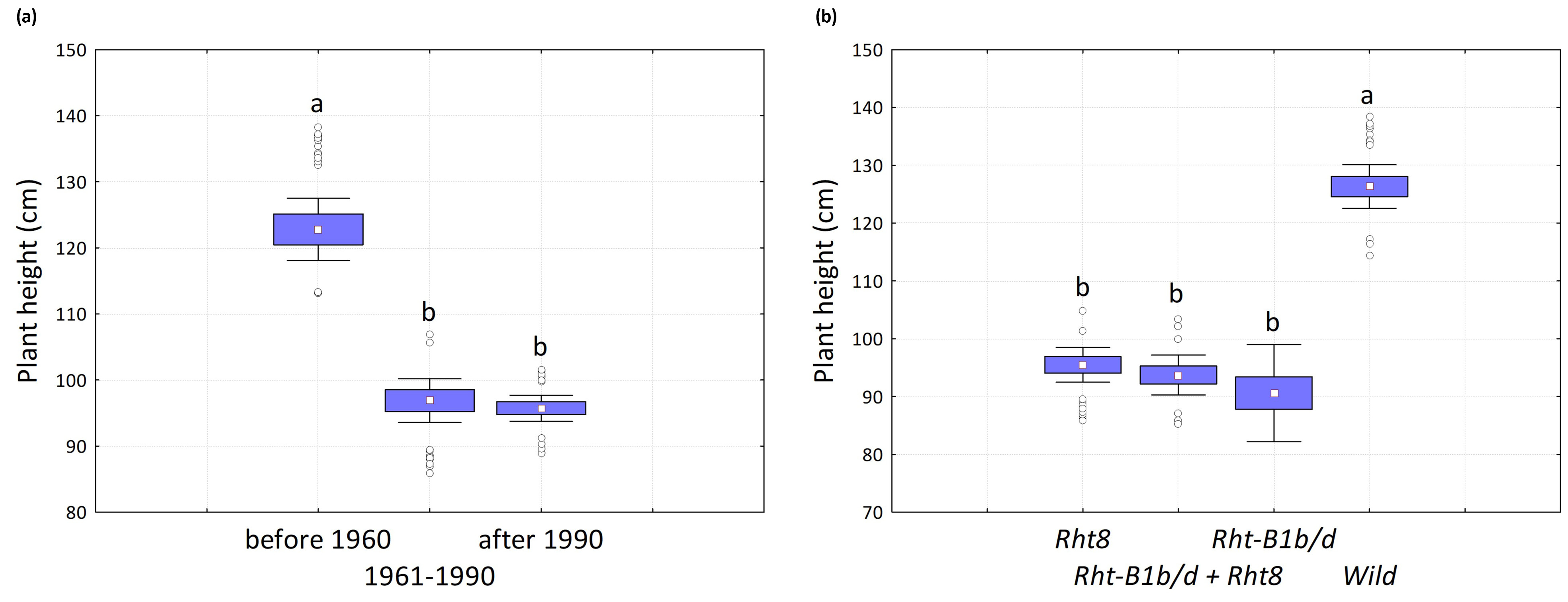

Supplement: Supplementary file 1 [file plants-13-02775-s001.zip › Figure_S2.jpg]

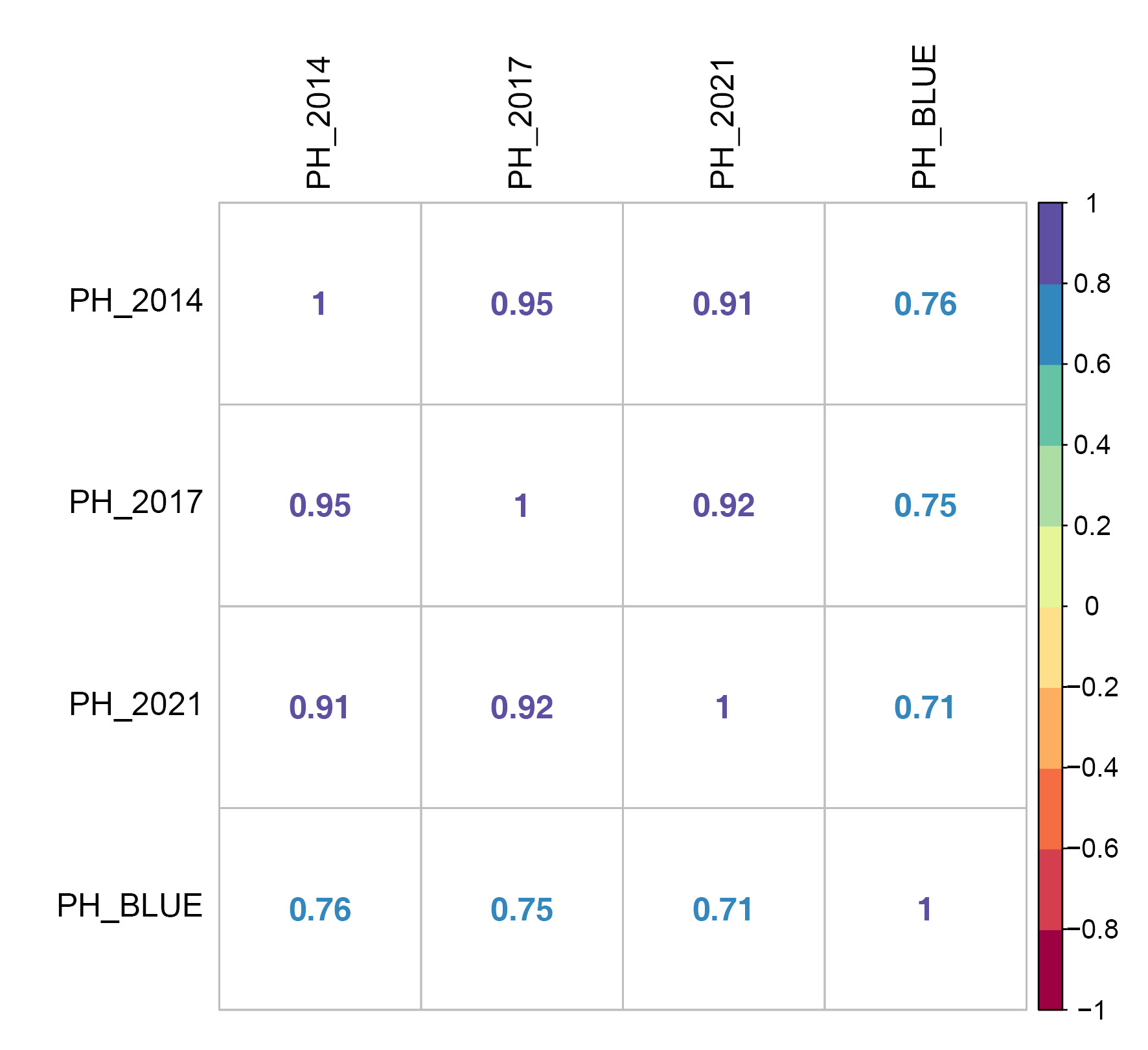

Supplement: Supplementary file 1 [file plants-13-02775-s001.zip › Figure_S3.jpg]

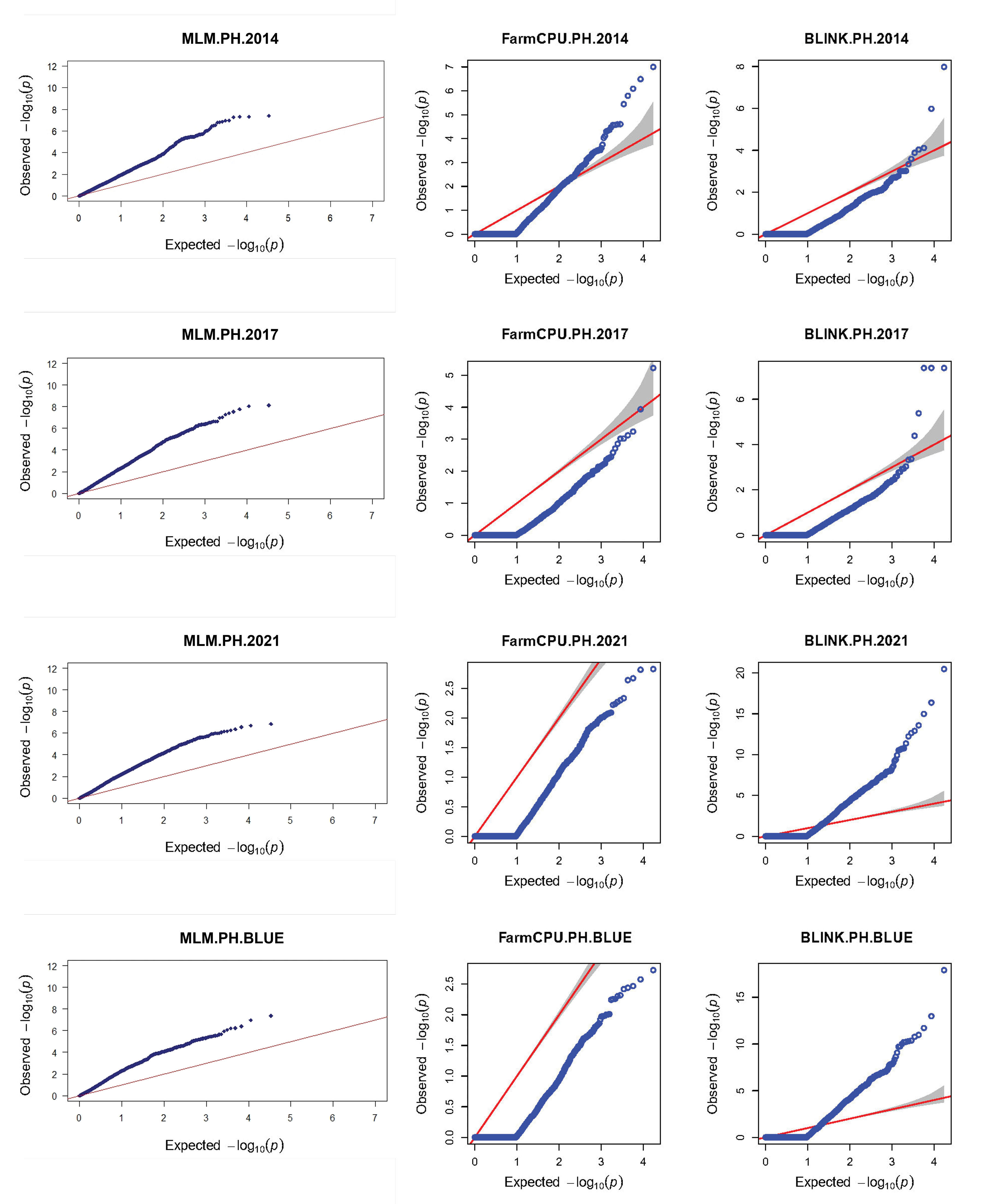

Supplement: Supplementary file 1 [file plants-13-02775-s001.zip › Figure_S4.jpg]
